# Exogenous WNT5A and WNT11 proteins rescue CITED2 dysfunction in mouse embryonic stem cells and zebrafish morphants

**DOI:** 10.1038/s41419-019-1816-6

**Published:** 2019-08-05

**Authors:** João M. A. Santos, Leonardo Mendes-Silva, Vanessa Afonso, Gil Martins, Rui S. R. Machado, João A. Lopes, Leonor Cancela, Matthias E. Futschik, Agapios Sachinidis, Paulo Gavaia, José Bragança

**Affiliations:** 1 Department of Biomedical Sciences and Medicine, University of Algarve, 8005-139 Faro, Portugal; 2 Centre for Biomedical Research (CBMR), University of Algarve, Campus of Gambelas, Building 8, room 2.22, 8005-139 Faro, Portugal; 3 Centre of Marine Sciences (CCMAR), University of Algarve, 8005-139 Faro, Portugal; 4 ABC—Algarve Biomedical Centre, 8005-139 Faro, Portugal; 5 School of Biomedical Sciences, Faculty of Medicine and Dentistry, Institute of Translational and Stratified Medicine (ITSMED), University of Plymouth, Plymouth, PL6 8BU UK; 6 Institute of Neurophysiology and Center for Molecular Medicine Cologne (CMMC), University of Cologne (UKK), Robert-Koch-Str. 39, 50931 Cologne, Germany

**Keywords:** Differentiation, Differentiation, Disease model, Disease model

## Abstract

Mutations and inadequate methylation profiles of *CITED2* are associated with human congenital heart disease (CHD). In mouse, *Cited2* is necessary for embryogenesis, particularly for heart development, and its depletion in embryonic stem cells (ESC) impairs cardiac differentiation. We have now determined that *Cited2* depletion in ESC affects the expression of transcription factors and cardiopoietic genes involved in early mesoderm and cardiac specification. Interestingly, the supplementation of the secretome prepared from ESC overexpressing CITED2, during the onset of differentiation, rescued the cardiogenic defects of *Cited2*-depleted ESC. In addition, we demonstrate that the proteins WNT5A and WNT11 held the potential for rescue. We also validated the zebrafish as a model to investigate *cited2* function during development. Indeed, the microinjection of morpholinos targeting *cited2* transcripts caused developmental defects recapitulating those of mice knockout models, including the increased propensity for cardiac defects and severe death rate. Importantly, the co-injection of anti-*cited2* morpholinos with either CITED2 or WNT5A and WNT11 recombinant proteins corrected the developmental defects of Cited2-morphants. This study argues that defects caused by the dysfunction of *Cited2* at early stages of development, including heart anomalies, may be remediable by supplementation of exogenous molecules, offering the opportunity to develop novel therapeutic strategies aiming to prevent CHD.

## Introduction

Despite clinical improvements and efforts to sensitize the population about heart disease risk factors and habits for prevention, the global burden of cardiovascular disease has increased worldwide^1^. Congenital heart disease (CHD) affects nearly 1% of new-borns and results from structural and functional cardiac anomalies occurring during pregnancy due to genetic and/or environmental causes. Significant progress in the comprehension of genetic causes has already been achieved through basic and clinical cardiovascular research^2,3^. Genetic screens of large cohort of patients worldwide have led to the identification of non-synonymous and synonymous variants in *CITED2* sequence associated with sporadic non-syndromic CHD^4–10^. In mouse models, *Cited2* knockout embryos died in utero with cardiac abnormalities resembling those commonly observed in children with CHD^11–19^. Ventricular (VSD) and atrial (ASD) septal defects, transposition of the great arteries and the tetralogy of Fallot (TOF) are the most frequent heart anomalies associated with *CITED2* variants in humans. Remarkably, most of the missense mutations clustered in the serine rich junction (SRJ) domain which is unique to CITED2^20^. Most of CITED2 variants identified in patients with CHD, only marginally affect the capacity of CITED2 to repress HIF-1α transcriptional activity and/or to co-activate TFAP2C transcription factor ex vivo^4–10^. *CITED2* mutations in patients may also result in severe impairment of the vascular endothelial growth factor (*VEGF*) and *PITX2C* expression which are, respectively, involved in normal development of the vasculature, and left-right patterning, as well as heart development^8^. *CITED2* variants identified within the 5ʹ and 3ʹ-untranslated regions associated with ASD, VSD, and TOF^4^, may result in abnormal stability, processing, localization or translation of the *CITED2* transcripts, and consequently decrease CITED2 protein expression. In addition, reduced *CITED2* expression levels and an abnormal hypermethylation of CpG islands at its promoter have also been correlated with CHD^7^. Thus, like in mouse embryos in which *Cited2* is haplodinsufficient^13^, a reduced expression of functional CITED2 during gestation may result in CHD in humans.

During gastrulation, the expression of *Brachyury* marks all cells of the mesoderm, then *Mesp1* is subsequently expressed in early cardiac mesoderm cells, which will specify into cardiac cells of the first and second heart fields that contribute to the bulk of the adult heart^21^. In mouse embryos, *Cited2* deletion in either BRACHYURY-expressing or MESP1-expressing cells did not overtly affect heart development and viability^11^, arguing that *Cited2* may play a role in cardiogenesis before mesoderm specification. Accordingly, mouse embryonic stem cells (ESC) are only markedly impaired for cardiac specification when *Cited2* is depleted at the onset of differentiation^22^. *Cited2* depletion at day 2 of differentiation, before mesodermal specification, has a marginal effect on cardiogenesis, while its depletion after mesoderm specification from day 4 of differentiation onwards, fails to overtly affect cardiac differentiation^22^. *Cited2-*depletion at the onset of differentiation downregulated the expression of early mesoderm and cardiogenic specifying genes, such as *Brachyury*, *Mesp1*, *Isl1*, *Gata4*, *Nkx2.5*, *Tbx5*, and *Nfat3*^22,23^. Heart development and ESC differentiation towards cardiac cell lineages also rely on the sequential and cooperative modulation of signaling pathways, such as the canonical and non-canonical Wnt, bone morphogenetic proteins (Bmp), Activin/Nodal, Fibroblast growth factors (Fgf), Notch and Hedgehog pathways. The Wnt/β-catenin canonical pathway is essential to exit the pluripotency state and for mesoderm specification, and its subsequent inhibition is required for the transition of mesoderm cells to cardiac progenitors and cardiomyocytes^24,25^. The biphasic regulation of the Wnt/β-catenin signaling pathway is orchestrated by secreted Frizzled‐related protein (sFRP) and Dickkopf1 (DKK1) antagonists, and by the action of Wnt non-canonical proteins, such as WNT5A and WNT11^24–26^.

To better understand the role of *Cited2* in early cardiogenic program, we employed microarray technology to identify the global transcriptional changes associated with *Cited2*-depletion at the onset of ESC differentiation. The analysis of gene expression profiles suggested a delay in mesoderm commitment and cardiac specification in *Cited2*-depleted cells, as well as the suboptimal expression of many modulators of signaling pathways and secreted factors critical for cardiogenesis. We show that the cardiogenic defects of *Cited2*-depleted ESC are amendable by supplementation of conditioned medium obtained from ESC overexpressing CITED2 or treatment with recombinant WNT5A and WNT11 proteins at the onset of differentiation. Finally, we provide evidence that CITED2, WNT5A and WNT11 recombinant proteins have the potential to rescue the developmental defects, including heart defects, and mortality caused by *cited2* depletion in zebrafish embryos.

## Materials and methods

### Zebrafish rearing, embryo generation and microinjection of morpholinos

The night before injection, adult zebrafish (AB) males and females were set up in breeding tanks separated by dividers to increase total egg production. Next morning, males and females were allowed to mate naturally and embryos were immediately collected after fertilization, and microinjected at one-cell stage with 4.6 nl containing 5 ng (~0.7 pmol) of the custom designed *Cited2* Morpholino CCATCATGCGGTCTACCATTCCC with a 3'-Carboxyfluorescein end modification which targeted the translational start site (UAG MO), a *cited2* Morpholino AACTTTGTAACCTTTACCTCTCCGC with 3'-Lissamine end modification targeting the splicing site in the exon 1 (SPLICING MO), or a combination of both 2.5 ng UAG MO and 2.5 ng of SPLICING MO prepared in 1× Danieau solution (58 mM NaCl, 0.7 mM KCl, 0.4 mM MgSO_4_, 0.6 mm Ca(NO3)_2_, and 5.0 mM HEPES, pH 7.6). Similarly, a standard non-targeting oligonucleotide CCTCTTACCTCAGTTACAATTTATA (Control MO) was also microinjected to control the procedures. All morpholinos were acquired from GeneTools. Zebrafish embryos were kept in embryo medium (E3 medium supplemented with penicillin/streptomycin to a final concentration of 10 units/ml penicillin and 100 µg/ml streptomycin) at 28.5 °C with a photoperiod of 14 h light and 10 h dark. The medium was changed every day and the dead embryos counted and discarded. For heart rate determination, the number of heart beats were counted from 10 s videos of hearts acquired from randomly picked embryos at 48 hpf. Live imaging and photography were captured on a Leica MZ 7.5 stereomicroscope (Leica Microsystems). All experiments were conducted in accordance with the regulation of the Directive 2010/63/EU (EU, 2010).

### Embryonic stem cells, culture conditions production of conditioned medium, and transient transfections

C2^Δ/Δ^[LA11], C2^fl/fl^[Cre] and E14/T mouse ESC lines were previously described, and were cultured on gelatine-coated plates in undifferentiating medium supplemented with LIF^22,27^. Differentiation was induced using the hanging-drop method in medium containing 10% FBS without LIF supplementation (differentiation medium) as previously described^22^, except otherwise indicated. For conditioned media production, undifferentiated E14/T ESC transfected with an episomal vector expressing a flag-tagged human CITED2 (E14T/flagCITED2) or the pPyCAGIP control vector (E14T/Control) were cultured at a density of 3 × 10^4^ cells/cm^2^ in undifferentiating conditions on 0.1% gelatine-coated 100 mm culture plates for 24 h as described elsewhere^22,27^. Next, cells were washed twice with PBS1× and subsequently cultured in plain GMEM medium (no supplements added) at 37 °C in a 5% CO_2_ incubator for 16 h. Resulting conditioned media collected either from E14T/flagCITED2 cultures (termed CM-CITED2 hereafter) or E14T/Control cultures (termed CM-Ctl hereafter) was filtered through 0.45 μm. For transient transfections E14/T cells were co-transfected with the with Wnt11-luc reporter plasmid (harboring a 1064 bp fragment of mouse *Wnt11* proximal promoter region) described elsewhere^28^, with either the flag-CITED2 expressing plasmid or the control vector pPyCAGIP, as previously described^27^.

### In vitro and in vivo rescue experiments

For rescue experiments, CM-Ctl or CM-CITED2 was mixed to the differentiation medium described above in a 1:1 proportion with 10% FBS final. The mix differentiation media were used only at the onset of C2^fl/fl^[Cre] ESC differentiation (Day 0, D0) for 48 h, concomitantly with the supplementation of 4 hydroxytamoxifen (4HT) at 1 μM final concentration or ethanol used as vehicle. The rest of the differentiation process was performed as described above. For the rescue experiments using Wnt5a-depleted or Wnt11-depleted CM-CITED2, 1.5 ml of conditioned medium, obtained from E4T/flagCITED2 cells prepared as described above, was incubated either with 5 μg of either rat monoclonal anti-Wnt5a (MAB645, R&D Systems) or rabbit polyclonal anti-Wnt11 (SC-50360, Santa Cruz) antibodies at 4 °C for 16 h. Next, the immunocomplexes were purified using 30 μl (50% wt/vol) Protein G sepharose beads suspension (GE Healthcare), pre-blocked with 1% BSA solution added to CM-CITED2 and incubated at 4 °C with agitation for 1 h. The immunocomplexes bound to the protein G sepharose beads were centrifuged out and used for western blotting as described below, while the remaining media CM-CITED2 depleted from Wnt5a or Wnt11 was used in C2^fl/fl^[Cre] ESC differentiation assays. Alternatively, secreted Wnt5a or Wnt11 proteins in the CM-CITED2 were blocked during differentiation by adding either 5 μg/ml of rat monoclonal anti-Wnt5a (MAB645, R&D Systems) or 2.5 μg/ml of rabbit polyclonal anti-Wnt11 (SC-50360, Santa Cruz) antibodies to the differentiation medium of C2^fl/fl^[Cre] ESC during the first 48 h of differentiation. The control experiments were performed with no addition of any antibody or by addition of 5 μg/ml of rabbit anti-IgG.

Mouse recombinant Wnt5a (rWnt5a) protein (GF146, Millipore) and human recombinant Wnt11 (rWnt11) protein (6179-WN, R&D Systems) reconstituted in PBS1× with 0.1% BSA, were supplemented either individually at 100 ng/ml or in combination at 50 ng/ml each, at the onset of C2^fl/fl^[Cre] ESC treated with 4HT or C2^Δ/Δ^[LA11] ESC differentiation for 48 h. For rescue of Zebrafish defects, the indicated amounts of rWnt5a and rWnt11 were microinjected at one-cell stage concomitantly with anti-Cited2 morpholinos. The 8R-CITED2 recombinant protein (full-length human CITED2 protein fused at its N-terminal domain to 8 arginines) production and purification was described elsewhere^22^. Prior to microinjection of 8R-CITED2 at one-cell stage concomittantly with anti-Cited2 morpholinos, the 8R-CITED2 protein samples were dialyzed using SnakeSkin Dialysis Tubing 3.5 k MWCO (Thermofisher), against PBS1X at 4 °C for 16 h to remove the phenylmethylsulfonyl fluoride (PMSF) serine protease inhibitor used during its production. 400 or 500 pg (~13.5 or 16.7 fmol) of 8R-CITED2 were microinjected as indicated.

### ESC differentiation and microarray data generation

The differentiation of C2^fl/fl^[Cre] ESC was performed by the hanging drop method in the presence of 1 μM 4HT or ethanol supplemented at the onset of the process as described previously^22^. The assessment and counts of beating foci in culture plates were performed as previously described^22^. For microarray studies, undifferentiated C2^fl/fl^[Cre] ESC (treated with ethanol for 48 h, corresponding to the onset of differentiation-D0) and C2^fl/fl^[Cre] ESC differentiated by hanging drop/embryoid bodies formation until D4 after ethanol or 4HT treatment for 48 h at the onset of differentiation were collected. RNA preparation, microarray hybridization to Mouse Genome 430 2.0 arrays (Affymetrix), as well as quality control of matrices, raw data background correction, summary and normalization of microarray data, and a PCA to evaluate transcriptomic variability between the samples obtained from ESC at D0 and differentiated cells at D4 upon treatment of ethanol or 4HT were performed as previously described^29^. Differential expression was determined through application of an empirical Bayes linear model, and the significance of the changes was adjusted using Benjamini–Hochberg method, with adjusted *p* < 0.05 considered statistically significant. After standardization of signal values to a mean of 0 and standard deviation (SD) of 1 significant probe sets were analyzed by *k*-mean clustering based on Euclidian distance measurement and *k* = 7 using the Cluster 3.0 tool. Gene ontology (GO) and KEGG pathway analysis was conducted using Enrichr^30,31^, and top enriched biological process terms were presented.

### Protein preparation, immunoprecipitation and western blotting

The immunocomplexes bound to the protein G sepharose beads obtained from CM-CITED2 and CM-Ctl as described above, were washed 5 times with PBS1× and immunopurified proteins were analyzed by western blot as previously described^27^, with a rat monoclonal anti-WNT5A (MAB645, R&D Systems) or rabbit polyclonal anti-WNT11 (SC-50360, Santa Cruz) antibodies used at 1:1000 and 1:200 dilution, respectively. Loading was monitored by staining of PVDF membrane-bound proteins by ponceau s. Western blotting assays performed using 15 μg of protein extracts from zebrafish embryos harvested at 24 and 48 hpf after microinjection of either combined AUG and SPLICING (2.5 ng of each morpholino) or non-injected the control embryos (Control) prepared using conditions previously described^27^. Proteins were separated by SDS-PAGE on TGX Stain-Free™ FastCast™ Acrylamide Kit (Bio-Rad). CITED2 protein was detected in zebrafish extracts using an anti-CITED2 rat monoclonal IgG_2A_ antibody (MAB5005, R&D System) used at 1:500 dilution as recommended by the manufacturer. Loading control was monitored by detection of total stained proteins transferred to the PVDF membrane.

### Quantitative real-time PCR (qPCR)

Total RNA isolation, complementary DNA synthesis and qPCR assays were carried out as previously described^22,27^, with primers listed in Supplemental Table S3.

### Statistical analysis

Statistical significance was determined via two-tailed Student’s t tests assuming unequal variance or using Fisher’s exact test of independence for more than 2 nominal variables, as indicated. *p* values < 0.05 were considered statistically significant.

## Results

### Delayed mesoderm commitment of *Cited2*-depleted cells

**Fig. 1:**
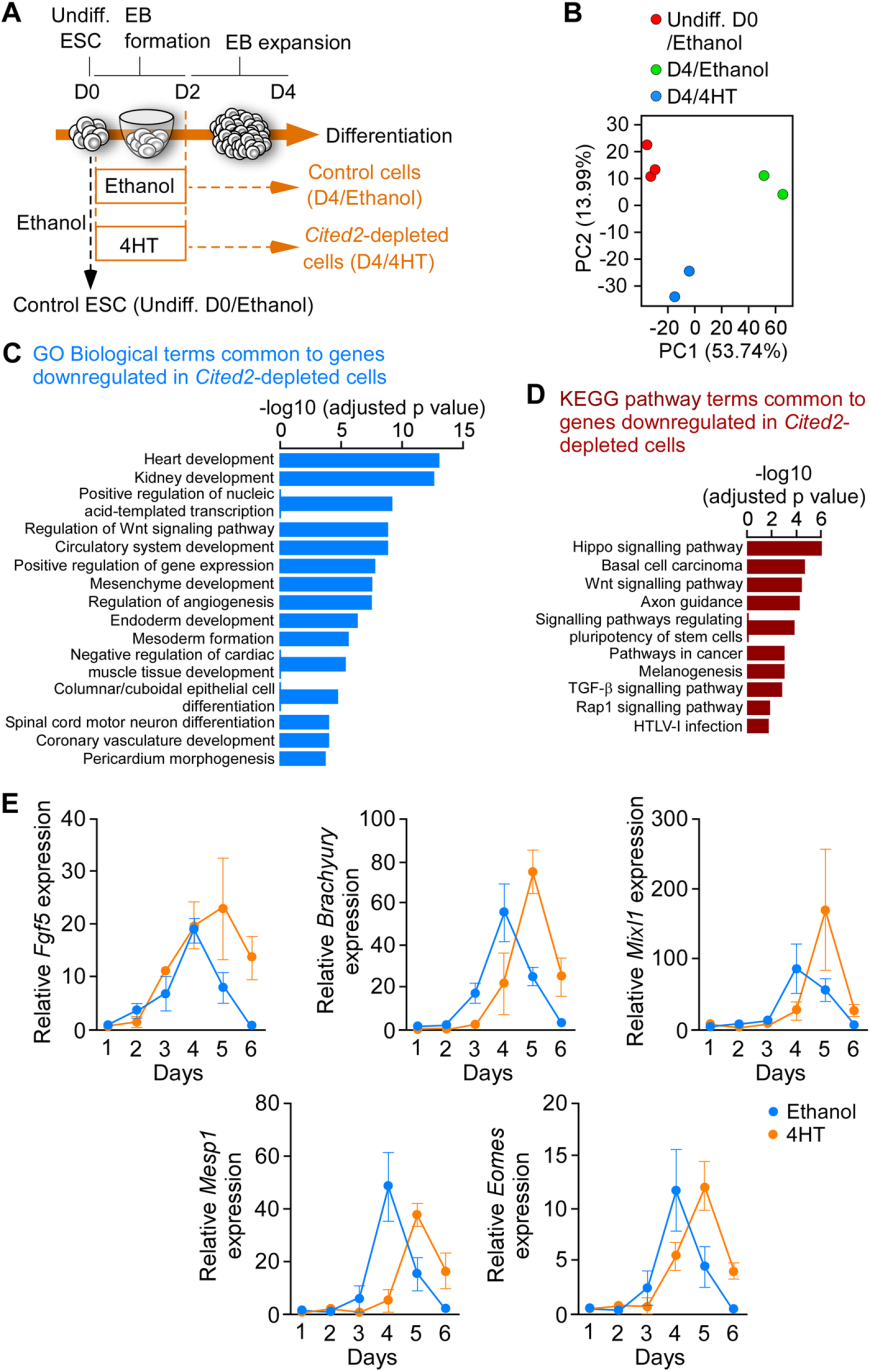
Cited2 depletion in embryonic stem cells delays mesoderm commitment. **a** Timeline depicting the protocol (embryoid bodies (EB) formation) used for differentiation C2^fl/fl^[Cre]ESC from D0 to D4. The time of ethanol or 4HT treatment is indicated. Undifferentiated control ESC (Undiff.) were treated with ethanol for 2 days. **b** Principal Component Analysis (PCA) of the entire normalized array datasets. After normalization of the entire transcriptome dataset obtained from undifferentiated C2^fl/fl^[Cre]ESC treated with ethanol (Undiff. D0/Ethanol) and differentiated for 4 days upon treatment with ethanol (D4/Ethanol) or 4HT (D4/4HT) for the first 48 h. Each sphere represents and individual sample. PC1 shows the main variability among the transcriptome differences and PC2 shows the second largest variability. **c** Top15 gene ontology biological process terms for the genes down-regulated by *Cited2* depletion at D4 of differentiation determined using Enrichr. **d** Top10 KEGG pathway terms for the genes downregulated by Cited2 depletion at D4 of differentiation determined using Enrichr. **e** Expression of the epiblastic (*Fgf5*) and mesoderm (*Brachyury, Mixl1, Mesp1, and Eomes*) markers from D1 to D6 of differentiation in cells generated from C2^fl/fl^[Cre] ESC treated with ethanol or 4HT as described in **a**. Results are presented as the mean ± SEM of three independent biological experiments

To investigate the mechanisms underlying the cardiogenic defects of *Cited2*-depleted ESC, we compared gene expression profiles at day 4 (D4) of differentiation of cells derived from C2^fl/fl^[Cre] ESC treated at the onset of differentiation for 48 h either with 4-hydroxytamoxifen (4HT) to deplete *Cited2* or with ethanol (vehicle), as previously described^22^. The gene expression profile of undifferentiated C2^fl/fl^[Cre] ESC (D0) treated for 48 h with ethanol was also established (Fig. 1a). The principal component analysis indicated that gene expression patterns of cells at D0, control and *Cited2*-depleted cells at D4 of differentiation are distinct (Fig. 1b). The analysis of gene expression profiles with a significant differential expression (adjusted *p* value < 0.05) and a positive or negative log2 fold-change >1.2 was performed between samples. Comparison of differentially expressed genes (DEGs) between control cells at D0 and D4 of differentiation was performed (Table S1). In addition, the functional enrichment analysis for these DEGs using Enrichr^30,31^, indicated that many upregulated genes at D4 of differentiation are related to cardiovascular development, and epithelial-to-mesenchymal transition (EMT) which is an early event of ESC commitment to mesoderm (Fig. S1). Fewer genes were differentially expressed between ESC at D0 and *Cited2*-depleted cells at D4 of differentiation (Table S2). The analysis of DEGs between *Cited2*-depleted and control cells at D4 of differentiation, revealed 263 upregulated and 334 downregulated genes (Table S2). Functional enrichment analysis for genes upregulated in *Cited2*-depleted cells at D4 of differentiation did not pinpoint any biological process with statistical significance (Table S2). However, this set contains genes associated with pluripotency, such as *Nanog*, *Klf2*, *Klf4*, *Klf5*, *Zfp42/Rex1*, *Sox2*, *Esrrb*, *Nr0b1*, *Dppa5a*, and *Zfp462* (Table S2), arguing for a delayed or restrained differentiation potential of *Cited2*-depleted ESC. We also compared the set of genes upregulated in *Cited2*-depleted cells with publicly available transcriptomics datasets related to stem cells using StemMapper^32^. This analysis confirmed that upregulated genes at D4 of differentiation in *Cited2*-control cells distinguish between different cell lineages (Fig. S1c). Compared to controls, *Cited2* depletion results in the loss of distinction between pluripotent, cardiac and neural lineages, suggesting further that *Cited2*-depleted cells are less differentiated. Interestingly, profiles of blood cell lineages remained distinct for *Cited2*-depleted and control cells. The analysis of biological processes enriched terms for genes downregulated in *Cited2*-depleted cells at D4 of differentiation indicated that *Cited2* regulates the expression of genes critical for many aspects of cardiovascular, kidney and endoderm development, mesoderm formation and angiogenesis (Fig. 1c and Table S2). Notably, *Brachyury*, *Mesp1*, *Isl1*, and *Gata4* identified in this set of genes were previously described to be affected by *Cited2* depletion in ESC^22,23^. To further validate the microarray data, we established a time course of expression for genes associated with pluripotency (*Dppa5a*), epiblast state/ectoderm (*Fgf5*), primitive streak/mesendoderm specification (*Brachyury*, *Mixl1*, *Mesp1*, *Eomesodermin (Eomes)*, *Cer1*, and *Gsc*) and secreted molecules (*Bmp4*, *Bmp5*, and *Wnt3)* promoting ESC differentiation (Fig. 1e and Fig. S2a). Overall, we confirmed that *Brachyury, Mixl1, Mesp1, Eomes*, and *Cer1* expression was decreased in *Cited2*-depleted cells at D4 of differentiation. However, the time course analysis from D1 to D6 of differentiation indicated that their expression peaked at D5 in *Cited2*-depleted cells, which is a day later than control cells. Moreover, at D5-D6 of differentiation *Fgf5*, *Brachyury*, *Mixl1*, *Mesp1*, *Eomes*, *Cer1*, and *Gsc* expression remained higher in *Cited2*-depleted cells. Together, our results suggest that *Cited2* depletion delayed mesoderm differentiation.

### *Cited2* depletion affects the expression of cardiopoietic factors

The analysis of enriched pathway terms also suggested that *Cited2* depletion affected the expression of modulators of signaling pathways normally under a tight regulation to instruct cell fate decisions^25^. Indeed, *Bmp2*, *Bmp5*, *Bmp7*, *Bmper*, *Fgf3, Fgf8*, and *Fgf10* expression was downregulated in *Cited2*-depleted cells, while *Fgf4* expression was increased (Fig. 1c, d and Table S2). In addition, several genes encoding for secreted or membrane-bound proteins which are either antagonists of the Wnt canonical pathway such as *Frzb*, *Dkk1*, *Wnt5a*, *Cer1*, *Apcdd1* or agonists such as *Rspo3*, *Wnt2*, *Wnt2b*, and *Wnt3* were amongst the most affected genes by *Cited2-*depletion (Table S2). Thus, cardiogenic defects of *Cited2*-depleted ESC may also result from the dysregulation of these signaling pathways.

**Fig. 2:**
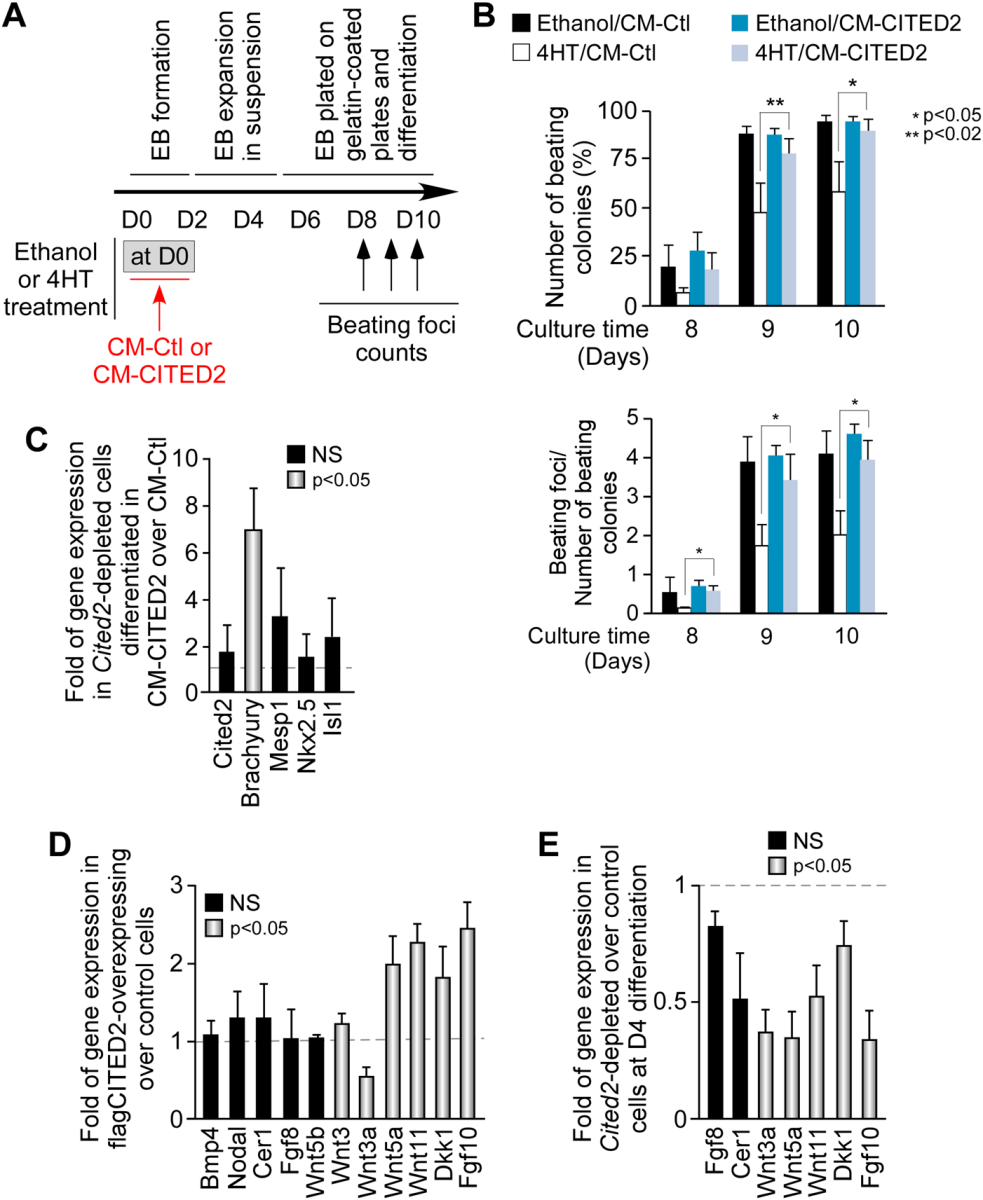
The secretome of CITED2 overexpression embryonic stem cells rescues cardiac differentiation of *Cited2*-depleted cells. **a** Timeline depicting the protocol used for differentiation C2^fl/fl^[Cre]ESC from D0 onward. The time of ethanol or 4HT treatment, as well as the supplementation with the conditioned media, and the days of beating activity assessment are indicated. **b** Percentage of colonies with contractile foci (top panel) counted at 8, 9 and 10 days after the initiation of differentiation in cell cultures derived from C2^fl/fl^[Cre] ESC treated with ethanol or 4HT at D0 of differentiation, and simultaneously supplemented with conditioned medium either from control cells (Ethanol/CM-Ctl and 4HT/CM-Ctl, respectively) or from cells overexpressing CITED2 (Ethanol/CM-CITED2 and 4HT/CM-CITED2, respectively). The number of beating foci per beating colony is also indicated (bottom panel). **c** Relative expression of *Cited2*, *Brachyury*, *Mesp1*, *Nkx2.5*, and *Isl1* determined by qPCR at D4 of differentiation in cultures derived from C2fl/fl[Cre] ESC treated with 4HT either in the presence of and CM-Ctl or CM-CITED2 as described in **b**. The expression of the indicated genes is presented as the fold of expression in cells treated with CM-CITED2 over cells treated with CM-Ctl. The black bars indicate variations without reaching statistical significance, and gray bars indicate genes with statistical significance by Student’s t-test. NS, not significant. **d** Relative expression of the indicated genes encoding secreted proteins involved in cardiogenesis, determined by qPCR in E14/T ESC transfected with a plasmid expressing flag-tagged CITED2 (flagCITED2) or the control empty plasmid (control cells). The results are presented as in **c**. **e** Expression of the indicated genes encoding secreted proteins involved in cardiogenesis, in cells treated as described in **c**. The results are presented as in **c**. Results are presented as the mean ± SEM of three independent biological experiments

To investigate whether CITED2 promoted the secretion of cardiopoietic factors, we transfected as previously described^22,27^, undifferentiated E14/T ESC with a control (E14T/Control) or flag-CITED2 expressing plasmid (E14T/flagCITED2). Next, these ESC were maintained for 16 h in medium deprived of LIF and serum (to prevent confounding effects of cardiogenic factors contained in the serum) for the preparation of conditioned medium (CM) enriched with ESC-secreted proteins. CM collected either from E14T/Control (CM-Ctl) or E14T/flagCITED2 (CM-CITED2) cultures were used to supplement the differentiation medium of C2^fl/fl^[Cre] ESC (Fig. 2). CM was supplemented at the onset of differentiation for 48 h, concomitantly with 4HT to deplete Cited2 or ethanol treatment (Fig. 2a). As expected, the number of beating which are indicative of cardiomyocyte spontaneous contractile activity, was reduced in cell cultures derived from *Cited2*-depleted ESC differentiated in 4HT/CM-Ctl in comparison to control cells (Ethanol/CM-Ctl), particularly at D9–D10 (Fig. 2b). Noticeably, not only the number of EB with spontaneous contractile activity was reduced in cells differentiated with 4HT/CM-Ctl, but the number of beating foci per EB was also decreased (Fig. 2b). Remarkably, the number of beating foci was similar in cultures derived from *Cited2*-depleted ESC differentiated in 4HT/CM-CITED2 and cultures of control conditions (Ethanol/CM-Ctl). CM-CITED2 supplementation did not alter the number of beating foci emerging in cells normally expressing *Cited2* (Ethanol/CM-Ctl compared to Ethanol/CM-CITED2). Interestingly, the supplementation of CM-CITED2 to *Cited2*-depleted cells stimulated *Brachyury* expression at D4 of differentiation without affecting significantly the expression of *Cited2*, *Mesp1*, *Nkx2.5* and *Isl1* (Fig. 2c). Thus, molecules secreted by ESC overexpressing flag-CITED2 may restore normal differentiation process of *Cited2*-depleted cells as early as mesoderm specification in a *Cited2*-independent manner.

### WNT5A and WNT11 restore normal cardiac differentiation of *Cited2*-depleted cells

To identify potential cardiopoietic factors secreted to the medium upon overexpression of flag-CITED2, we assessed *Wnt3*, *Wnt3a*, *Wnt5a*, *Wnt5b*, *Wnt11*, *Dkk1*, *Bmp4*, *Nodal*, *Cerl1*, *Fgf8*, and *Fgf10* expression by qPCR in E14T/flagCITED2 and E14T/Control ESC (Fig. 2d). These genes are essential for early mesoderm induction and cardiac specification, encoding modulators of Wnt, Bmp, Tgfβ, and Fgf signaling pathways^25^. No significant differences were observed in *Bmp4*, *Nodal* and *Wnt5b* expression (Fig. 2d). The analysis of microarray data also indicated that *Fgf8* and *Cer1* expression may be sensitive to *Cited2* depletion (Table S2). However, only a mild decrease of *Fgf8* and *Cer1* expression without statistical significance was observed by qPCR (Fig. 2e). In contrast, *Wnt5a*, *Wnt11*, *Dkk1*, and *Fgf10* expression was significantly increased in cells overexpressing CITED2, while *Wnt3* expression was only marginally altered and *Wnt3a* expression reduced (Fig. 2d). The downregulation of *Wnt3a*, *Wnt5a*, *Wnt11*, *Dkk1*, and *Fgf10* in *Cited2*-depleted cells at D4 of differentiation observed in the microarray data was confirmed by qPCR (Fig. 2e and Table S1). Thus, *Wnt5a*, *Wnt11*, *Dkk1*, and *Fgf10* expression patterns correlated with *Cited2* expression (Fig. 2d, e), arguing for a positive effect of *Cited2* on the expression of these genes. A direct involvement of CITED2 in their transcriptional regulation remains to be established. Together, these observations suggested that the delay in *Cited2-*depleted ESC differentiation may result from a defective expression of secreted factors, such as Wnt5a, Wnt11, and Dkk1.

**Fig. 3:**
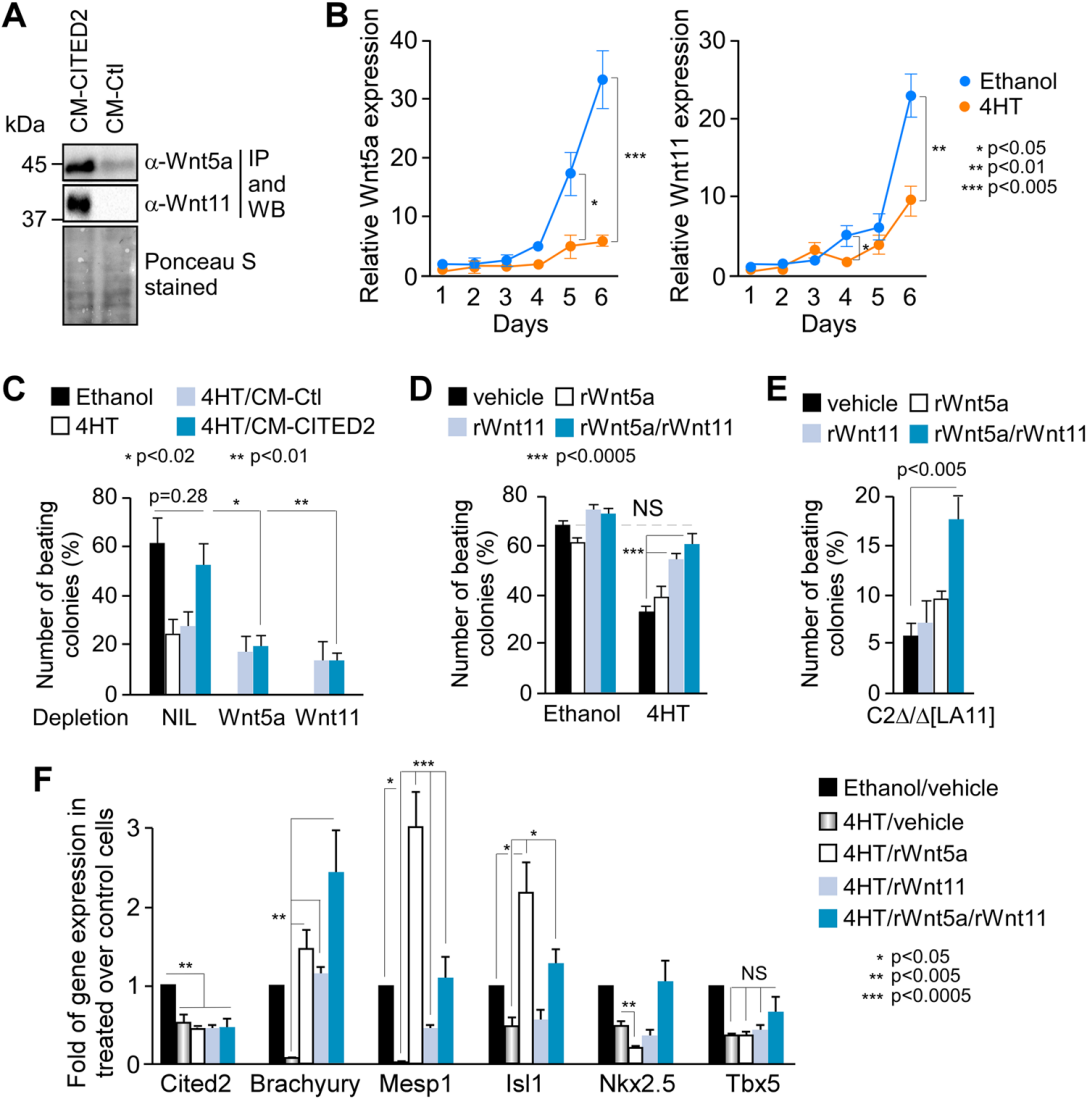
WNT5A and WNT11 proteins have the potential to surpass *Cited2* lack of function in cardiac differentiation. **a** Conditioned media obtained from E14/T ESC transfected with the flagCITED2 expressing vector (CM-CITED2) or the control plasmid (CM-Ctl) immunoprecipitated (IP) with anti-WNT5A and anti-WNT11 antibodies. Ponceau S stained blots on 10% of the input material used for immunoprecipitation show loading controls. **b** Time course of *Wnt5a* and *Wnt11* expression determined by qPCR in samples prepared as described in Fig. 1d. **c** Percentage of colonies with contractile foci counted at D10 of differentiation in cell cultures derived from C2^fl/fl^[Cre] ESC treated with ethanol or 4HT at D0 of differentiation, or with 4HT in differentiation medium supplemented with conditioned medium either from control cells (4HT/CM-Ctl) or from cells overexpressing CITED2 (4HT/CM-CITED2), or 4HT/CM-CITED2 medium depleted from WNT5A, WNT11 depletion by immunoprecipitation as described in **a**, or no depletion using PBS 1× as vehicle (NIL). **d** Percentage of colonies with contractile foci counted at D8 of differentiation in cell cultures derived from C2^fl/fl^[Cre] ESC treated with ethanol or 4HT at the onset of differentiation (vehicle), in the presence of 100 ng/ml of recombinant WNT5A (rWnt5a) or WNT11 (rWnt11) proteins or simultaneously with rWnt5a and rWnt11 (50 ng/ml of each). **e** Percentage of colonies with beating foci derived from C2^Δ/Δ^[LA11] ESC at D10 of differentiation, in presence of rWnt5a or/and rWnt11, or PBS 1x as described in **d**. **f** Relative expression of *Cited2*, *Brachyury*, *Mesp1*, *Isl1*, *Nkx2.5*, and *Tbx5* determined by qPCR at D4 of differentiation in cultures derived from C2^fl/fl^[Cre] ESC treated as indicated in **d**. Gene expression in ethanol/vehicle conditions was set to 1. Bars represent mean ± SEM of three independent experiments

To determine whether CM-CITED2 contained WNT5A and WNT11 proteins, and whether these proteins contributed to restore the cardiogenic potential of *Cited2*-depleted ESC, we performed a western blotting with plain CM-CITED2 and CM-Ctl using specific antibodies against WNT5A or WNT11. However, no proteins were detected, suggesting that these molecules were either absent or in very low quantity in the CM. To enrich the western blotting samples with WNT5A and WNT11, proteins from CM-CITED2 and CM-Ctl were immunoprecipitated using either WNT5A or WNT11 specific antibodies. Immunopurified proteins were analyzed by western blotting, which revealed that Wnt5a and Wnt11 proteins were more abundant in CM-CITED2 than CM-Ctl (Fig. 3a). In addition, *Wnt5a* and *Wnt11* transcripts expression was markedly impaired in *Cited2*-depleted cells during differentiation (Fig. 3b). Concordantly, we showed that the overexpression of flag-CITED2 in E14/T cells stimulated the activity of *Wnt11* promoter (Fig. s2b), arguing that *Wnt11* expression may be under the direct control of CITED2 regulation. Next, we used CM-CITED2 and CM-Ctl depleted either from WNT5A or WNT11 proteins by immunoprecipitation in differentiation assays (Fig. 3c). Unlike control CM-CITED2, WNT5A or WNT11 immuno-depleted CM-CITED2 failed to restore the emergence of beating foci to the level of control cells (Fig. 3c). Direct supplementation of antibodies against WNT5A or WNT11 to CM-CITED2 at the onset of differentiation also neutralized the rescue (Fig. S2c). Altogether, these observations indicated that secreted WNT5A and WNT11 proteins compensate for the loss of *Cited2* in early events of ESC cardiac differentiation. These observations were corroborated by the ability of recombinant WNT5A (rWnt5a) and WNT11 (rWnt11) proteins to complement the loss of *Cited2* function in ESC (Fig. 3d). Individual and combined supplementation of rWnt5a and rWnt11, at the onset of the process for 2 days, stimulated cardiomyocyte differentiation of *Cited2*-depleted ESC. However, only the co-supplementation of rWnt5a and rWnt11 fully restored the differentiation efficiency to the level of control cells. Supplementation of rWnt5a and rWnt11 had no significant effect on the cardiac differentiation of control cells (Fig. 3d). Interestingly, rWnt5a/rWnt11 treatment also significantly stimulated the cardiogenic potential of *Cited2*-null C2^Δ/Δ^[LA11] ESC (Fig. 3e). Therefore, the potential of rWnt5a/rWnt11 to rescue the cardiogenic program of *Cited2*-depleted ESC is independent of endogenous CITED2 expression/function. Accordingly, the treatment of *Cited2*-depleted C2^fl/fl^[Cre] ESC with rWnt5a and rWnt11 did not affect *Cited2* expression levels in depleted cells (Fig. 3f). Interestingly, *Mesp1* and *Isl1* expression, strongly downregulated in *Cited2*-depleted cells, was restored to levels comparable to those of control cells by the combined treatment with rWnt5a/rWnt11 (Fig. 3f). On the other hand, *Brachyury* expression was higher in *Cited2*-depleted cells treated with rWnt5a/rWnt11 than in control cells, while *Nkx2.5* and *Tbx5* expression was not significantly altered in the same conditions. Noticeably, the treatment of *Cited2*-depleted cells either with only rWnt5a or rWnt11 restored normal *Brachyury* expression levels (Fig. 3f). On the other hand, *Mesp1* and *Isl1* expression exceeded the levels of control cells when treated solely with rWnt5a, and *Mesp1* expression levels were increased without reaching normal levels by rWnt11 treatment (Fig. 3f). Interestingly, WNT5A and WNT11 homodimers have been reported to physically interact, and their combined exogenous expression synergizes to regulate normal *Xenopus* embryonic patterning, while the expression of either WNT5A or WNT11 alone inhibit the endogenous Wnt pathways^33^. Therefore, the combined supplementation of rWnt5a/rWnt11 may balance the action of these proteins to adequately regulate *Brachyury*, *Mesp1*, and *Isl1* expression during ESC differentiation.

### WNT5A and WNT11 restores viability and cardiogenesis to Cited2-morphants

**Fig. 4:**
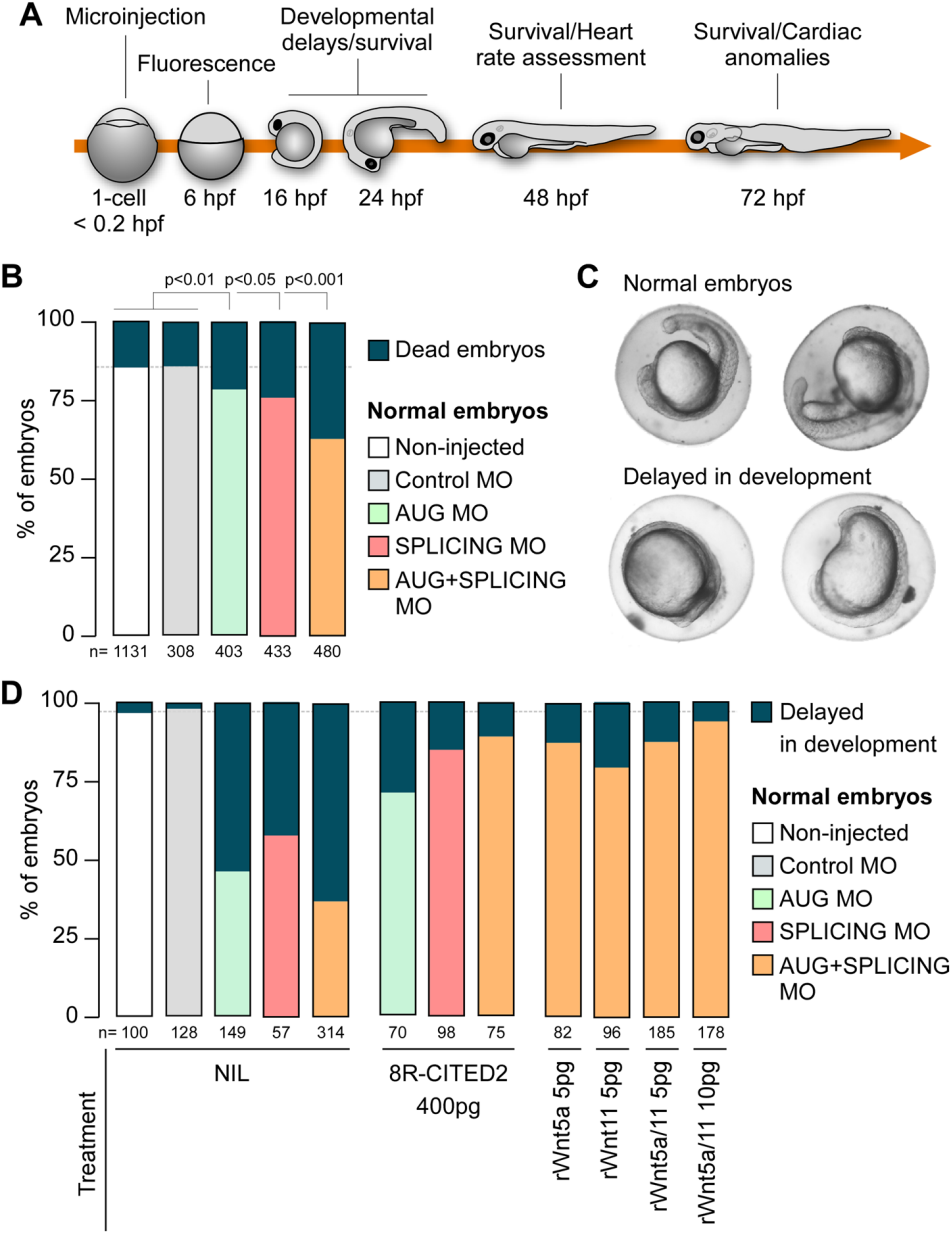
Early developmental defects of Cited2 morphants are reverted by WNT5A and WNT11. **a** Schematic representation of the experimental steps and analysis performed with zebrafish embryos. The timings of development are indicated in hours post fertilization (hpf). **b** Percentage of embryos which are normal or dead at 24 hpf, after injection of 5 ng (0.7 pmol) of control morpholino (Control MO), anti-*cited2* morpholinos targeting either the transcriptional start site (AUG MO; 5 ng) or the splicing site in the exon 1 (SPLICING MO; 5 ng), simultaneously with AUG MO and SPLICING MO (AUG+SPLICING MO, 2.5 ng of each morpholino), as well as non-injected embryos (Non-injected). Statistical significance was determined against control embryos using Student’s *t* test. **c** Brightfield images of live embryos showing the representative morphological features of zebrafish embryos considered as normal (top panel) or delayed in development (bottom panel) at 20 hpf. **d** Percentage of embryos which are normal or delayed in the developmental process at 20 hpf, after injection of morpholinos as described in **b**, in the presence of 400 pg of recombinant CITED2 protein (8R-CITED2), rWnt5a and rWnt11 alone (5 pg) or in combination (rWnt5a/11) in a final amount of 5 pg (2.5 pg each) or 10 pg (5 pg each), or no treatment (NIL). In panels, **b** and **d** *n* represents the number of embryos analyzed in each condition in at least 3 independent experiments

**Fig. 5:**
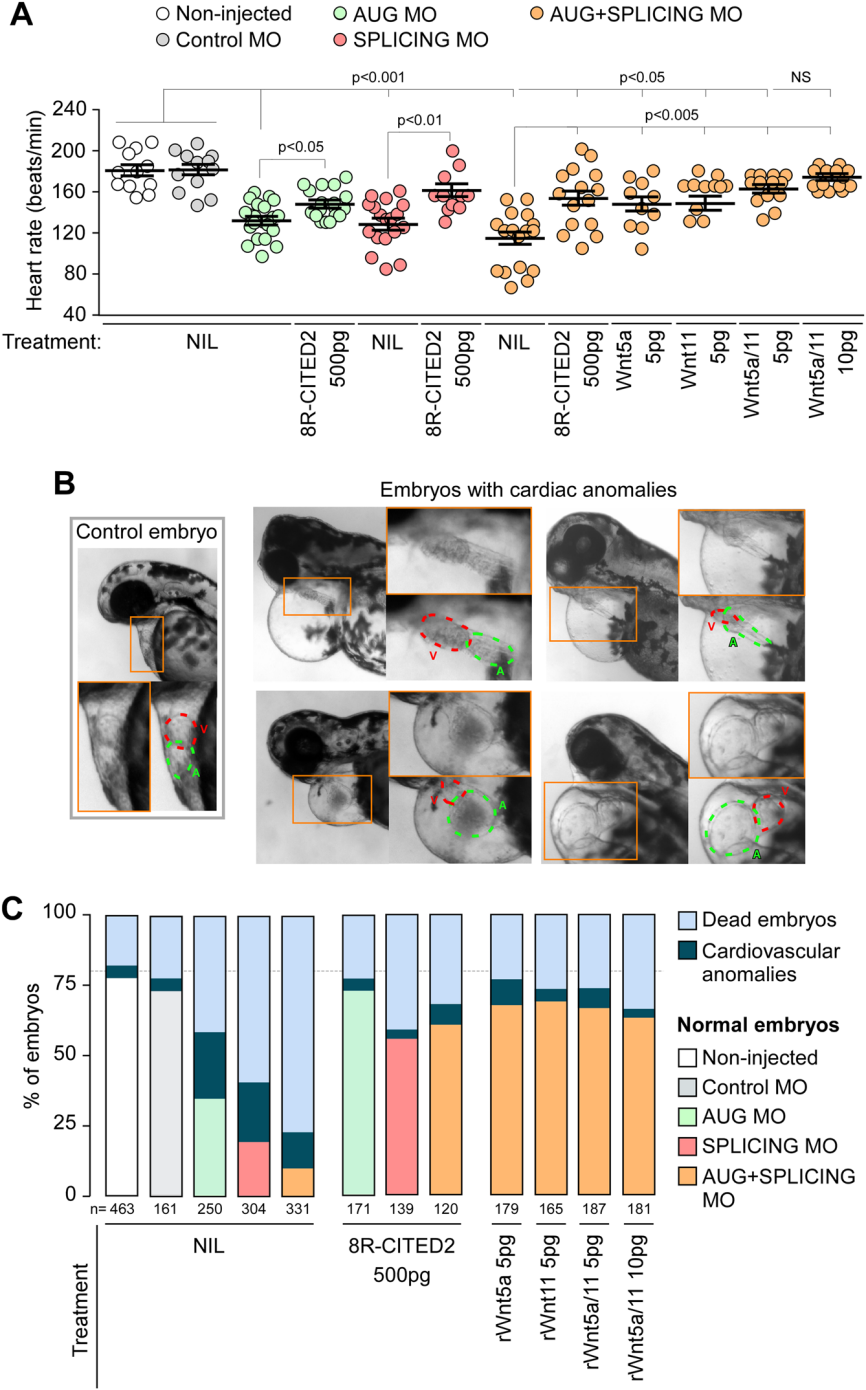
Supplementation of WNT5A and WNT11 to early embryos rescues the lethality and cardiac defects of Cited2 morphants. **a** Heart rate of embryos treated at presented in Fig. 4d measured at 48 hpf at 28 °C. Each dot represents the value obtain for a single embryo. The mean and standard deviations for each condition are also presented. Statistical significance was determined against control embryos using Student’s *t* test. **b** Brightfield images of live embryos showing a representative morphology at 48 hpf of control embryos and embryos affected with cardiac anomalies, such as pericardial effusion, linear heart tube, enlarged atrium and/or ventricle. **c** Percentage of embryos which are normal, dead or presenting cardiac anomalies at 72 hpf, after injection of morpholinos as described in Fig. 4d, with the exception that 500 pg of CITED2 recombinant protein were injected. The death rate presented is the cumulative death observed in each condition from 6 to 72 hpf. n represents the number of embryos analyzed in each condition in at least 3 independent experiments

We next sought to investigate whether the supplementation of Wnt5a and Wnt11 would rescue the developmental defects caused by Cited2 depletion in vivo. To this aim, we used zebrafish (*Danio rerio*), a widespread animal model to study cardiovascular development^34,35^. Zebrafish has a unique *cited2* gene which is expressed from pre-gastrulation stage and across later embryonic developmental stages^36,37^. We assessed the role of *cited2* in zebrafish development using specific antisense morpholinos targeting either the *cited2* mRNA translation start site (AUG-MO) or the splicing site in the exon 1 (SPLICING-MO) to deplete Cited2 by microinjection at one-cell stage (Fig. 4a). Depletion of Cited2 protein in embryos microinjected with AUG/SPLICING-MO was monitored by western blotting (Fig. S3a). Control animals were microinjected with a standard non-targeting morpholino (Control-MO). The development and viability of embryos microinjected with Control-MO did not differ significantly from non-injected embryos, indicating that the procedure was not originating developmental defects (Fig. 4b). Conversely, microinjection of AUG-MO and SPLICING-MO individually or in combination triggered the increase of embryonic lethality at 24 hpf in comparison to control embryos (Fig. 4b). Alive embryos microinjected with AUG-MO and SPLICING-MO individually or in combination, display more frequent signs of developmental delay than control animals (Fig. 4c, d). To evaluate whether the developmental defects observed in Cited2-morphants were specifically caused by loss of Cited2 expression, we performed co-microinjections of the morpholinos against *cited2* with recombinant human 8R-CITED2 protein. This protein which is able to cross the cellular membranes and reach the nucleus when applied to the culture medium^22^, significantly decreased the number of embryos displaying developmental delays (Fig. 4d). Thus, the developmental delays of Cited2-morphants were likely due to loss of Cited2 expression. At 48 hpf, living Cited2-morphants consistently developed bradycardia (~120–140 beats/min) in comparison to control embryos which presented a normal heart rate at ~170 beats/min (Fig. 5a). Decrease of heart rate is a sign of cardiac dysfunction which was further confirmed by the presence of pericardial effusion and cardiac anomalies such as linear heart tube, enlarged heart chambers or the presence of a virtually unique chamber in Cited2-morphants (Fig. 5b). At 72 hpf, the cumulative death rate of Cited2-morphants was significantly increased compared to control embryos, particularly in embryos microinjected with both anti-*cited2* morpholinos in which the mortality rate was ~75%. Concomitantly, Cited2-morphants that survived at 72 hpf displayed cardiovascular anomalies at higher levels than controls (Fig. 5c). In addition to cardiac abnormalities, Cited2-morphants often presented a delay in hatching, low mobility, back curvature and curved tail defects (Fig. s3f). Of interest, heart rate and cardiovascular defects, as well as lethality caused by anti-*cited2* morpholinos were partially rescued by the co-microinjection of 8R-CITED2, arguing further that the remarkable defects observed in the embryos were specific to Cited2 depletion.

Since we determined that the supplementation of rWnt5a and rWnt11 to ESC at the onset of differentiation restored the cardiogenic functions lost by *Cited2*-depletion, we assessed the ability of these proteins to rescue the defects of Cited2-morphant embryos. The micro-injection of rWnt5a and rWnt11 individually or in combination restored normal developmental timings, normal heart rate, and reduced the overall mortality and cardiovascular anomalies of Cited2-morphants (Figs. 4c, 5a, c). Although, the cumulative death of Cited2-morphants at 72 hpf was not restored to the level of control animals by the combined microinjection of rWn5a/rWnt11, surviving embryos presented less frequent heart defects (Fig. 5c). Of relevance, microinjection of rWnt5a/rWnt11 proteins alone without *cited2*-targeting MO did not affect the development of the embryos in our conditions (Fig. S3b–e).

Together our observations indicate that the critical loss of *Cited2* function at the onset of cardiac differentiation of ESC, or in the early steps of zebrafish development may be surpassed by treatment WNT5A and WNT11 proteins.

## Discussion

CITED2 function is critical for normal cardiac development in humans and mice. In this report, we establish that in comparison to control zebrafish embryos, Cited2-morphant have a high mortality rate and increased frequency of heart anomalies. Like for *Cited2*-null mouse embryos^6^, supplementation of human CITED2 recombinant protein to Cited2-morphants rescued the defects. Thus, zebrafish is a valid model to study the developmental functions of Cited2. Our observations support a critical role for CITED2 in cardiac development across distant vertebrate species.

Distinct subpopulations of *Brachyury-*expressing mesoderm cells emerge in a controlled temporal fashion during ESC differentiation and in vivo^38^. Unlike the specification of hematopoietic cells which may occur from all *Brachyury*-expressing cells arising from D2.5 to D4 of ESC differentiation, cells with cardiogenic potential emerge in a narrow temporal window at D3-D3.5 of differentiation^38^. Evidence from mouse models and in vitro ESC-based differentiation experiments had suggested that *Cited2* contributes to cardiac development at early stages of gastrulation or even during the specification of pluripotent cells to mesoderm^11,22^. During ESC differentiation *Cited2* expression is biphasic, as it declines at D0-D2 of differentiation and increases from D3 onwards^22^. Thus, *Cited2* expression at around D2-D3 of differentiation may be critical to optimally induce cardiac mesoderm specification. Here, we show that *Cited2*-depletion in ESC delays the expression of mesoderm markers by 24 h and prolonged the expression of the epiblastic marker *Fgf5*. The capacity of *Cited2*-depleted ESC to exit the pluripotency state upon differentiation may not be altered, since the downregulation of pluripotency-related genes expression in these cells was comparable to control ESC at D0-D3 of differentiation^22^. However, the microarray analysis we performed suggests that *Nanog*, *Klf2*, *Klf4*, *Klf5*, *Zfp42/Rex1*, *Sox2*, *Esrrb*, *Nr0b1*, *Dppa5a*, and *Zfp462* genes related to pluripotency remain expressed at higher levels at D4 of differentiation in *Cited2*-depleted cells. Thus, the delay of *Cited2*-depleted differentiation may be due to the stabilization of a transition state between primed pluripotent cells and mesoderm cells. A similar phenomenon was also observed with *Strip2*-depleted murine ESC^39,40^.

We have highlighted the importance of *Cited2* for optimal expression of cardiopoietic factors, such as *Wnt5a* and *Wnt11*. Interestingly, during mouse embryos gastrulation and ESC differentiation, the canonical Wnt pathway is required for the formation of the primitive streak and to initiate mesoderm specification. *Wnt5a* and *Wnt11* expression is important for these early developmental events through the stimulation of EMT^41^. Secretion of WNT5A, WNT11, and DKK1 is also essential to inhibit the canonical Wnt pathway and allow mesoderm cells to originate cardiac progenitors^25^. Interestingly, Wnt5a and Wnt11 are expressed during quail and zebrafish development at various stages, including the induction of mesoderm^26,42–45^. In human ESC, WNT5A, and WNT11, by activation of the non-canonical pathway, regulate cardiac differentiation through mesoderm induction, activation of MESP1 expression and cardiomyocyte differentiation^46^. Interestingly, the decrease of *Brachyury*, *Mesp1*, and *Isl1* expression observed in *Cited2*-depleted ESC was reverted close to normal levels by recombinant WNT5A and WNT11 treatment in a *Cited2*-independent manner. Thus, impaired cardiac differentiation of *Cited2-*depleted ESC may result from a defective inhibition of the canonical Wnt/β-catenin pathway or activation of non-canonical Wnt pathways causing low expression levels of *Brachyury*, *Mesp1*, and *Isl1*. Importantly, the supplementation of recombinant WNT5A and WNT11 proteins truly complemented CITED2 function in the cardiogenic process of *Cited2*-depleted ESC.

Finally, we provide evidence that the deleterious dysfunction of CITED2 in vivo, may be surpassed by the transient exposure of the embryos at very early stages of development with exogenous of proteins, such as WNT5A and WNT11. Like for *Cited2*, the function of a broader number of early cardiogenic genes may be complemented by treatment with exogenous molecules. Indeed, cardiac defects and mid-gestation lethality of *Id*-null mouse embryos were also partially rescued by intraperitoneal injection of ESC into mouse females prior to conception^47^. In those injected ESC, IGF1 and WNT5A proteins were proposed to be responsible for complete correction of the cardiac defects. It would be of interest to further characterize the secretome of CITED2-overexpressing ESC which may contain additional secreted factors or exosomes with properties to compensate for the dysfunction of genes required at early stages of cardiogenesis. The discovery of such molecules may facilitate the development of novel clinical strategies to limit or prevent CHD.

## Supplementary information

Supplemental Figures

Material Table Supp1 - DEGs

Material Table Supp2 - GO_Biological_andKEGG_2018Enrichr

Material Table Supp3
