# Supplementary material for: Exogenous WNT5A and WNT11 proteins rescue CITED2 dysfunction in mouse embryonic stem cells and zebrafish morphants
Source: Cell Death Dis. 2019 Aug 5;10(8):582. doi: 10.1038/s41419-019-1816-6 (PMC6680046; doi:10.1038/s41419-019-1816-6)
Supplement: Supplementary file 1 — Supplemental Figures [file 41419_2019_1816_MOESM1_ESM.docx]

Title: **Exogenous Wnt5a and Wnt11 proteins rescue Cited2 dysfunction in mouse embryonic stem cells and zebrafish morphants**

João M. A. Santos^1,2^, Leonardo Mendes-Silva^1,2¶^, Vanessa Afonso^1,2¶^, Gil Martins^4^, Rui S. R. Machado^1,2^, João A. Lopes^1,2^, Leonor Cancela^1,3,4^, Matthias E. Futschik^2,4,5^, Agapios Sachinidis^6^, Paulo Gavaia^1,4^ and José Bragança^1,2,3^*

**Supplementary Figures**

**
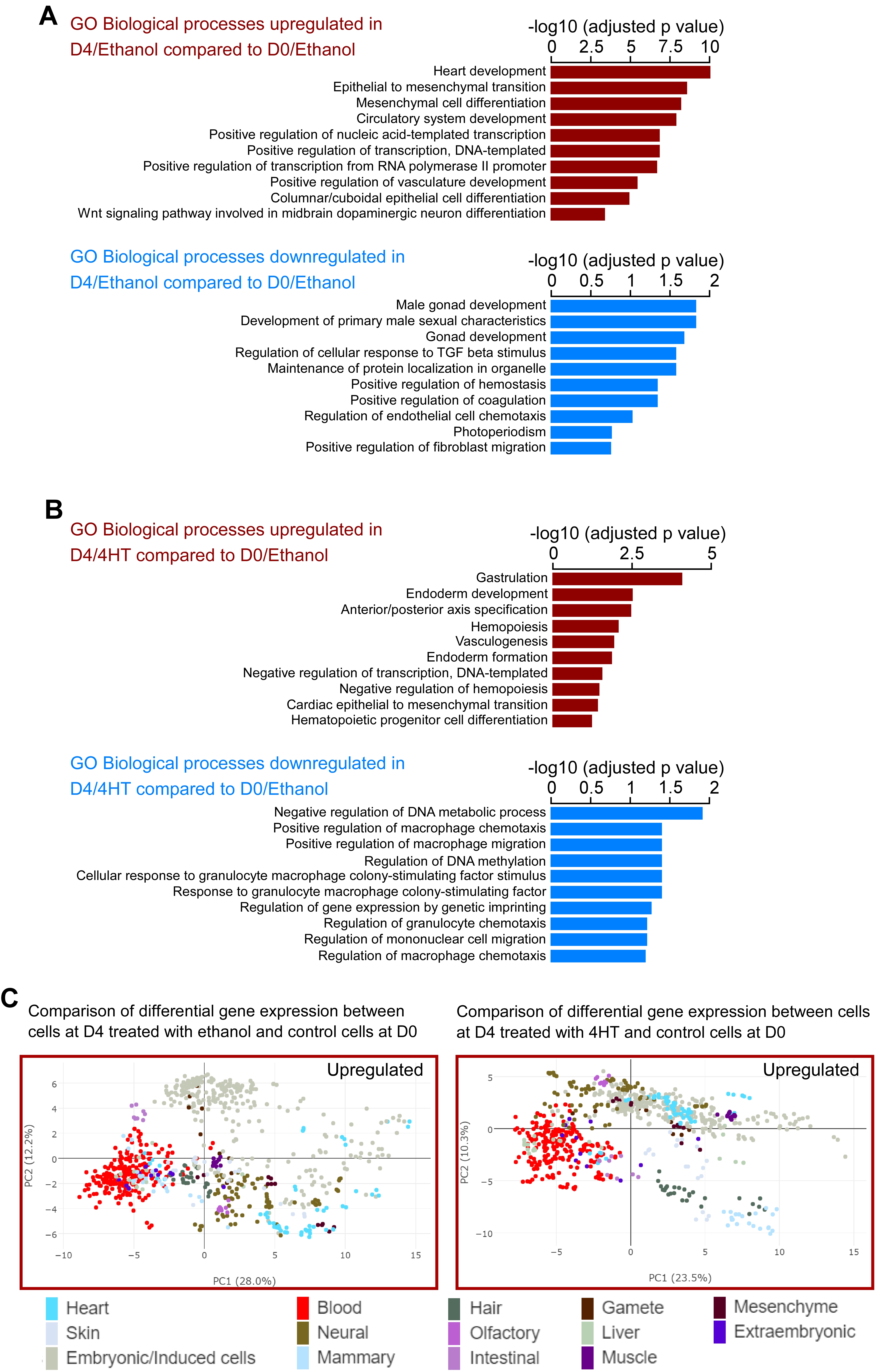
**

**Figure S1 - Cited2 depletion in embryonic stem cells alters biological processes.** (**A**) Top10 gene ontology biological process terms determined using Enrichr for genes either upregulated (top) or downregulated (bottom) between D0 and D4 of differentiation in control conditions. (**B**) Top10 gene ontology biological process terms determined using Enrichr for genes either upregulated (top) or downregulated (bottom) between D0 and D4 of differentiation in *Cited2*-depleted conditions. (**C**) Global comparison of the genes upregulated by *Cited2*-depletion in ESC with the gene profiles of other stem cells using StemMapper.

**
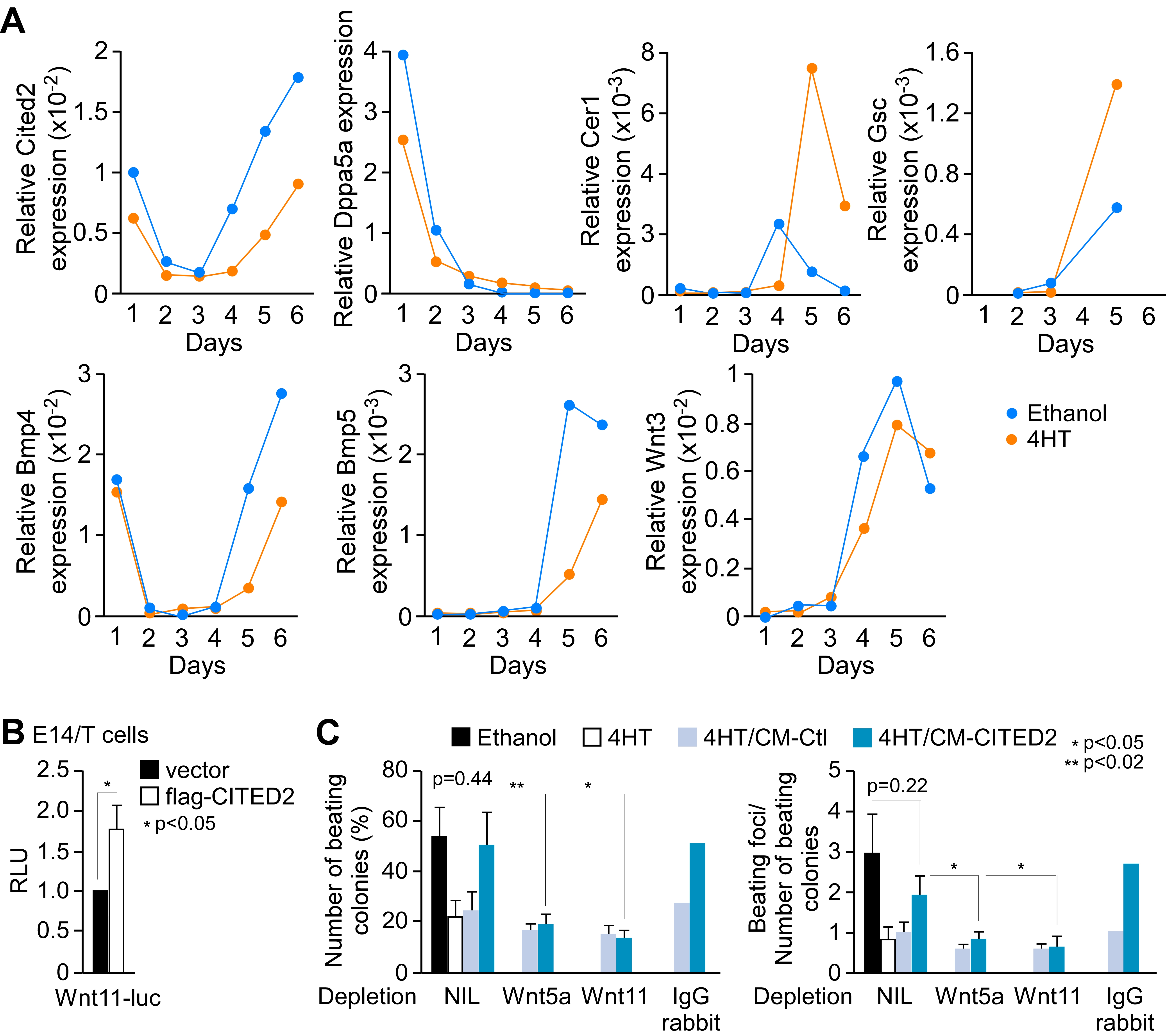
**

**Figure S2 – Neutralization of Wnt5a and Wnt11 proteins in conditioned medium impairs the recovery of cardiac differentiation in cells depleted from** ***Cited2*.** (**A**) Expression of the indicated genes from D1 to D6 of differentiation in cells generated from C2^fl/fl^[Cre] ESC treated with ethanol or 4HT as described in Figure 1D, and normalized to *gapdh* expression. Results are presented as the mean two independent biological experiments. (**B**) Wnt11‐luc activity in E14/T cells co-transfected with pPyCAGIP‐flagCITED2 (white bars) or pPyCAGIP (black bars). Results are presented as the mean ± SEM of three biological experiments. (**C**) Percentage of colonies with contractile foci counted at D10 of differentiation in cell cultures derived from C2^fl/fl^[Cre] ESC treated with ethanol or 4HT at D0 of differentiation, or with 4HT in differentiation medium supplemented with conditioned medium either from control cells (4HT/CM-Ctl) or from cells overexpressing CITED2 (4HT/CM-CITED2), or 4HT/CM-CITED2 medium in the presence of either anti-Wnt5a (5 μg/ml), anti-Wnt11 (2.5 μg/ml) or control rabbit IgG (5 μg/ml), or no depletion using PBS1X as vehicle (NIL).

**
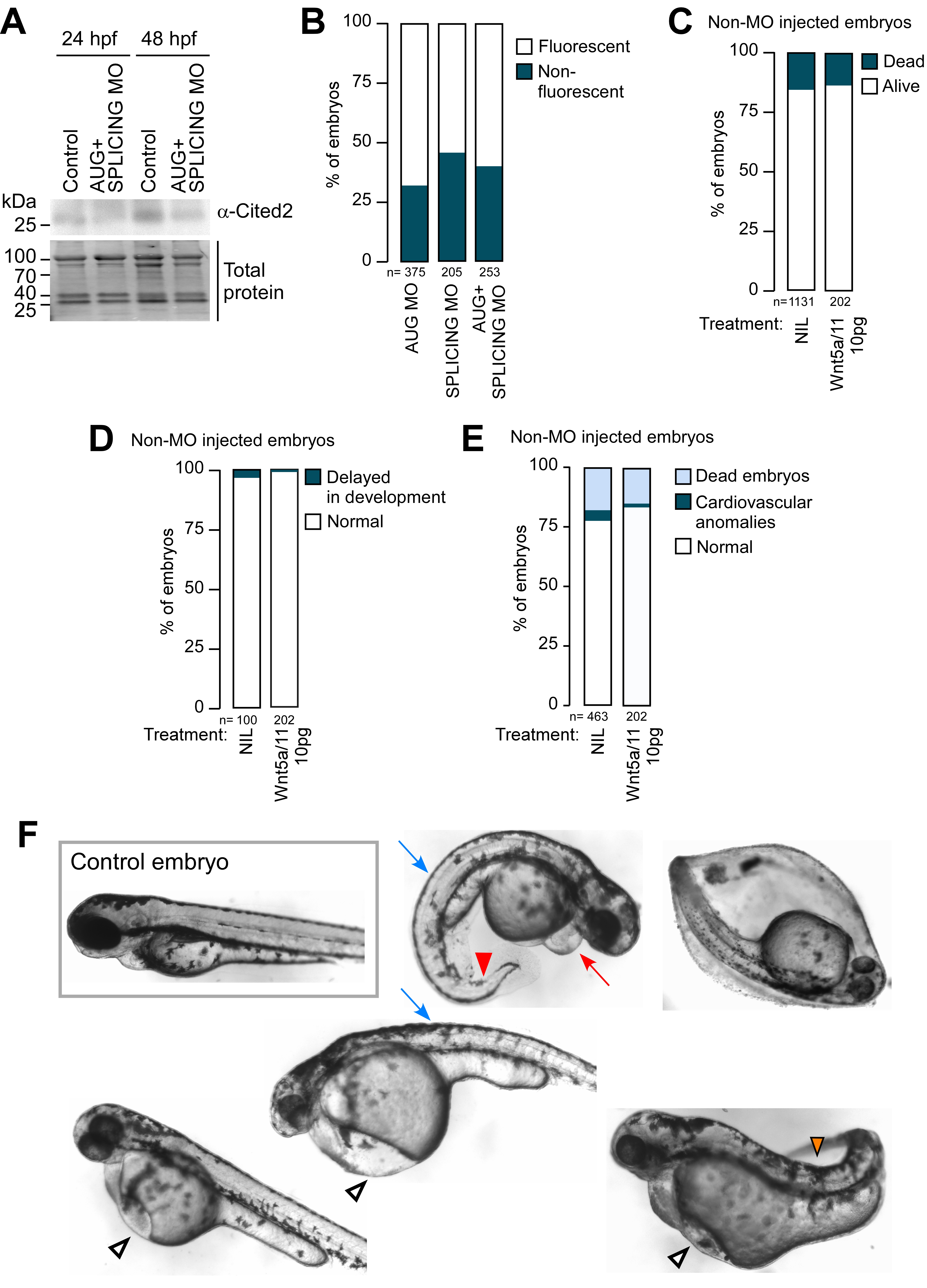
**

**Figure S3 – Cited2 morphants display developmental defects, but the sole microinjection of recombinant Wnt5a and Wnt11 does not affect the development of normal embryos.** (**A**) Top panel: Cited2 protein levels determined by western blotting in protein extracts from zebrafish embryos at 24 hpf and 48hpf after microinjection of either combined AUG and SPLICING (AUG+SPLICING MO, 2.5 ng of each morpholino) or control non-injected embryos (Control). Bottom panel: Total protein stained with TGX Stain-Free™ FastCast™ Acrylamide Kit (Bio-Rad) transferred on the PVDF membrane was used to control for loading. The position of the Cited2 protein is in agreement with the predicted molecular weight (~25 kDa). (**B**) Percentage of fluorescent embryos at 6 hpf and after the individual and combined microinjection of AUG MO (5 ng) and SPLICING MO (5ng) (AUG+SPLICING MO, 2.5 ng of each morpholino) at 1-cell stage. (**C**) Percentage of dead and live embryos at 24 hpf and after combined microinjection of rWnt5a and rWnt11 (5 pg of each protein) or no injection at 1-cell stage. (**D**) Percentage of live embryos presenting developmental delays at 24 hpf and after microinjection of rWnt5a and rWnt11 as described in B. (**E**) Percentage of dead and live embryos presenting a normal morphology or cardiac anomalies at 72 hpf and after microinjection of rWnt5a and rWnt11 as described in B. For all panels, n represents the number of embryos analysed in each condition in at least 2 independent experiments. (**F**) Brightfield images of live embryos showing the representative morphology at 72 hpf of control embryos and embryos injected with 5 ng of anti-Cited2 morpholinos as described in Figure 4B. The red arrow indicates pericardial edema, the blue arrow an abnormal curvature of the spine, the white arrowhead a slight swelling or edema of the of the yolk sac, the red arrowhead a curvy tail and the orange arrowhead a severe notochord defect. An embryo that fail to hatch is also represented.
