# Supplementary material for: Exogenous WNT5A and WNT11 proteins rescue CITED2 dysfunction in mouse embryonic stem cells and zebrafish morphants
Source: Cell Death Dis. 2019 Aug 5;10(8):582. doi: 10.1038/s41419-019-1816-6 (PMC6680046; doi:10.1038/s41419-019-1816-6)
Supplement: Supplementary file 4 — Material Table Supp3 [file 41419_2019_1816_MOESM4_ESM.pdf]

Table S3 – Primers used for qPCR

qPCR	Forward primer	Reverse primer	Reference
Bmp4	TTCCTGGTAACCGAATGCTGA	CCTGAATCTCGGCGACTTTTT	(Kim et al., 2010)
Bmp5	AGCCTGCAAGAAGCACGAAC	AAAAGAACATTCCCCGTCACAA	(Kim et al., 2010)
Brachyury	CTCTAATGTCCTCCCTTGTTGCC	TGCAGATTGTCTTTGGCTACTTTG	(Ivanova et al., 2006)
Cer1	CTCTGGGGAAGGCAGACCTAT	CCACAAACAGATCCGGCTT	(Li et al., 2013)
Cited2	CGCATCATCACCAGCAGCAG	CGCTCGTGGCATTCATGTTG	(Kranc et al., 2015)
Dkk1	CTGAAGATGAGGAGTGCGGCTC	GGCTGTGGTCAGAGGGCATG	(Kemp et al., 2005)
Dppa5a	GCCGTGCGTGGTGGATAAGC	GCCAAACAGATATTTCAGCACCAGC	(Tanaka et al., 2002)
Eomes	CCGGACAACTACGATTCCA	ACCTCCAGGGACAATCTGATG	(van den Ameele et al., 2012)
Fgf5	CTGTATGGACCCACAGGGAGTAAC	ATTAAGCTCCTGGGTCGCAAG	(Ivanova et al., 2006)
Fgf8	CCAGCCCCAAACTACCCCGAGGAG	CGCGCAGACCCAGCCCAGGAT	(Zhang et al., 2003)
Fgf10	CAGCGGGACCAAGAATGAAG	TGACGGCAACAACTCCGATTT	(Sultana et al., 2009)
Gapdh	TCCCACTCTTCCACCTTCGATGC	GGGTCTGGGATGGAAATTGTGAGG	(Ivanova et al., 2006)
Gsc	AAACGCCGAGAAGTGGAACAAG	AAGGCAGGGTGTGTGCAAGTAG	(Ivanova et al., 2006)
Isl1	CTTAAGCATGCCCTGTAGCTGG	CAGACAGGAGTCAAACACAATCCC	(Ivanova et al., 2006)
Mixl1	CGCCAGAGTGGGAAGTCA	CAGGGCAATGGAGGAAAAC	(Date et al., 2013)
Mesp1	TGTACGCAGAAACAGCATCC	TTGTCCCCTCCACTCTTCAG	(Zhong and Jin, 2009)
Nkx2.5	CCACTCTCTGCTACCCACCT	CCAGGTTCAGGATGTCTTTGA	(Sachinidis et al., 2006)
Nodal	TGGCGTACATGTTGAGCCTCT	TGAAAGTCCAGTTCTGTCCGG	(Ogawa et al., 2007)
Tbx5	GGACCCAGTCCCTTGAATGG	TCCAGGCTGAGGAGTTCTAGGC	(Ivanova et al., 2006)
Wnt3	ACCTGGAGAAGGCTGGAAGT	CTTGTCCTTGAGGAAGTCGC	(Sultana et al., 2009)
Wnt3a	TGGCTCCTCTCGGATACCTC	AAAGCTACTCCAGCGGAGGC	(Nemeth et al., 2007)
Wnt5a	CAAATAGGCAGCCGAGAGAC	TCTAGCGTCCACGAACTCCT	(Chen et al., 2007)
Wnt5b	AGATAGGTAGCCGAGAGACTGC	GGTAGCCGTACTCCACGTTG	(Choi et al., 2011)
Wnt11	GCTCCATCCGCACCTGTT	CGCTCCACCACTCTGTCC	(Sultana et al., 2009)

REFERENCES – Related to Table S3

Chen, Y., Haviernik, P., Bunting, K.D., and Yang, Y.-C. (2007). Cited2 is required for normal hematopoiesis in the murine fetal liver. Blood *110*, 2889-2898.

Choi, M., Jung, K., Park, J., Das, N., Chung, M., Choi, I., Lee, B., Park, K., and Chai, Y. (2011). Ethanol-induced small heat shock protein genes in the differentiation of mouse embryonic neural stem cells. Archives of Toxicology *85*, 293-304.

Date, Y., Hasegawa, S., Yamada, T., Inoue, Y., Mizutani, H., Nakata, S., and Akamatsu, H. (2013). Major amino acids in collagen hydrolysate regulate the differentiation of mouse embryoid bodies. Journal of Bioscience and Bioengineering *116*, 386-390.

Ivanova, N., Dobrin, R., Lu, R., Kotenko, I., Levorse, J., DeCoste, C., Schafer, X., Lun, Y., and Lemischka, I.R. (2006). Dissecting self-renewal in stem cells with RNA interference. Nature *442*, 533-538.

Kemp, C., Willems, E., Abdo, S., Lambiv, L., and Leyns, L. (2005). Expression of all Wnt genes and their secreted antagonists during mouse blastocyst and postimplantation development. Developmental Dynamics *233*, 1064-1075.

Kim, B., Kim, Y., Sakuma, R., Hui, C.-C., RÃ¼ther, U., and Jorgensen, J.S. (2010). Primordial germ cell proliferation is impaired in Fused Toes mutant embryos. Developmental Biology *349*, 417-426.
Kranc, K.R., Oliveira, D.V., Armesilla-Diaz, A., Pacheco-Leyva, I., Matias, A.C., Escapa, A.L., Subramani, C., Wheadon, H., Trindade, M., Nichols, J.*, et al.* (2015). Acute loss of Cited2 impairs Nanog expression and decreases self-renewal of mouse embryonic stem cells. Stem Cells *33*, 699-712.
Li, L., Liu, C., Biechele, S., Zhu, Q., Song, L., Lanner, F., Jing, N., and Rossant, J. (2013). Location of transient ectodermal progenitor potential in mouse development. Development *140*, 4533-4543.
Nemeth, M.J., Topol, L., Anderson, S.M., Yang, Y., and Bodine, D.M. (2007). Wnt5a inhibits canonical Wnt signaling in hematopoietic stem cells and enhances repopulation. Proceedings of the National Academy of Sciences *104*, 15436-15441.
Ogawa, K., Saito, A., Matsui, H., Suzuki, H., Ohtsuka, S., Shimosato, D., Morishita, Y., Watabe, T., Niwa, H., and Miyazono, K. (2007). Activin-Nodal signaling is involved in propagation of mouse embryonic stem cells. J Cell Sci *120*, 55-65.
Sachinidis, A., Schwengberg, S., Hippler-Altenburg, R., Mariappan, D., Kamisetti, N., Seelig, B., Berkessel, A., and Hescheler, J. (2006). Identification of small signalling molecules promoting cardiac-specific differentiation of mouse embryonic stem cells. Cell Physiol Biochem *18*, 303-314.
Sultana, D., Tomita, S., Hamada, M., Iwanaga, Y., Kitahama, Y., Khang, N., Hirai, S., Ohigashi, I., Nitta, S., Amagai, T*., et al.* (2009). Gene expression profile of the third pharyngeal pouch reveals role of mesenchymal MafB in embryonic thymus development. Blood *113*, 2976-2987.
Tanaka, T.S., Kunath, T., Kimber, W.L., Jaradat, S.A., Stagg, C.A., Usuda, M., Yokota, T., Niwa, H., Rossant, J., and Ko, M.S.H. (2002). Gene Expression Profiling of Embryo-Derived Stem Cells Reveals Candidate Genes Associated With Pluripotency and Lineage Specificity. Genome Research *12*, 1921-1928.
van den Ameele, J., Tiberi, L., Bondue, A., Paulissen, C., Herpoel, A., Iacovino, M., Kyba, M., Blanpain, C., and Vanderhaeghen, P. (2012). Eomesodermin induces Mesp1 expression and cardiac differentiation from embryonic stem cells in the absence of Activin. EMBO Rep *13*, 355-362.
Zhang, Y., Wang, S., Song, Y., Han, J., Chai, Y., and Chen, Y. (2003). Timing of odontogenic neural crest cell migration and tooth-forming capability in mice. Developmental Dynamics *226*, 713-718.
Zhong, X., and Jin, Y. (2009). Critical roles of coactivator p300 in mouse embryonic stem cell differentiation and nanog expression. J Biol Chem *284*, 9168-9175.
